# Supplementary material for: Growth zone segmentation in the milkweed bug Oncopeltus fasciatus sheds light on the evolution of insect segmentation
Source: BMC Evol Biol. 2018 Nov 28;18:178. doi: 10.1186/s12862-018-1293-z (PMC6262967; doi:10.1186/s12862-018-1293-z)
Supplement: Supplementary file 1 — Additional examples of embryonic and hatchling phenotype of the Of-slp, Of-hh and Of-odd RNAi experiments. All embryos are stained for the inv segmental marker. Younger embryos are in the left column, older embryos on the middle column, hatchlings on the right column. Embryos stages are not identical in all columns for all experiments, specimens in the same row are not linked. (PDF 14900 kb) [file 12862_2018_1293_MOESM1_ESM.pdf]

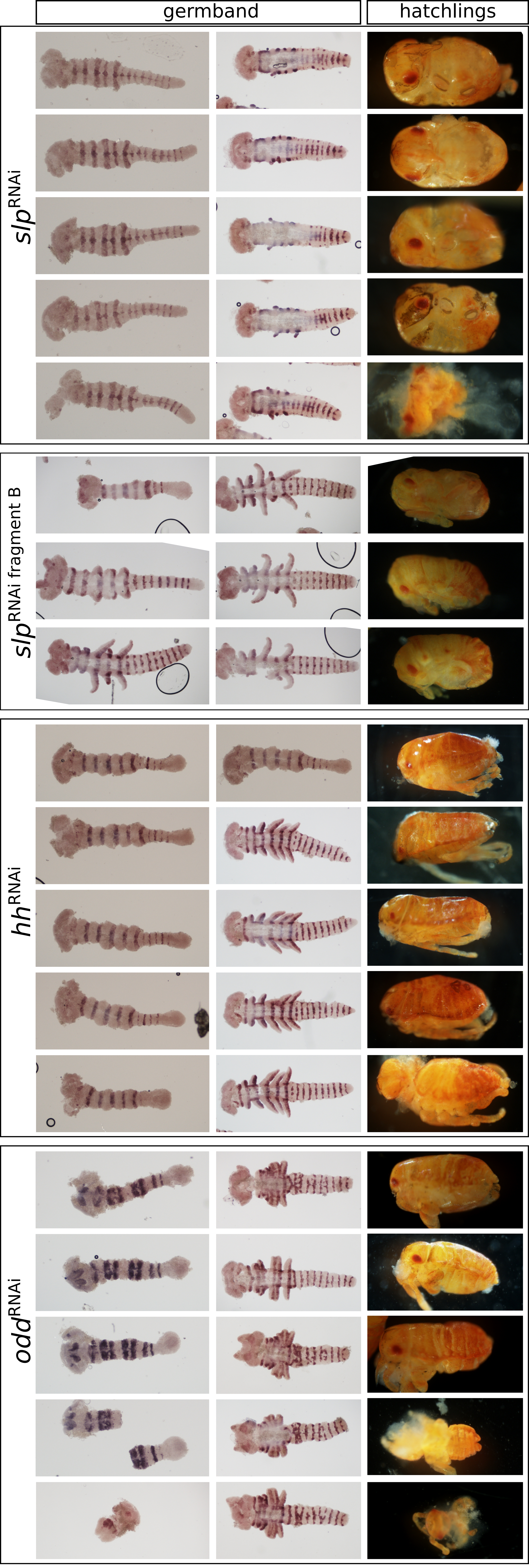

While the embryonic phenotypes of the *slp* RNAi embryos were consistent in relation to abdominal segmentation, some variation is observed in the intensity of the knock down on the appendages. In embryos where segmentation is almost at its end we find a lack of preliminary limb buds (*slp* RNAi, germband, left column). In later stages (*slp* RNAi, germband, middle column) almost all embryos completely lack the T2 appendages, and residual stumps are left form the T1 and T3 limbs. Hatchlings display a complete lack of appendages and severe truncation of mouthparts in addition to many more defects related to later embryonic processes such as dorsal closure etc.

The specificity of this phenotype is striking when examining the embryos of the second *slp* RNAi fragment, where almost no segmentation effect is seen, some “curling” is seen in T1 and T3 appendages- yet the effect on the T2 appendages is still very strong and specific.The *hh* RNAi embryos also show very little variation in the observed phenotype. A very mild phenotype is seen in the segmental borders. Interestingly, the strongest effect is consistently seen in the border between abdominal segments 1 and 2. This is seen best in the older embryos (middle column), but might be already observable in earlier stages (left column), where the corresponding stripe seems to be weaker than the following ones and might be related to the transition between blastoderm and germband modes of segmentation.

The main *hh* RNAi effect is observed in the hatchling head which is small and malformed. This too is already noticeable in the early embryos where the head structure lacks some of its typical folds. *odd* depleted embryos display abdominal segment defects, from slight segments border defects to segment fusion. Like the *slp* RNAi embryos, the most striking effect is seen in the appendages. Appendage fusion vary from “mild” where only some thoracic segments are fused, the most effected embryos where the all mouth and thoracic segments seem to be almost completely fused. This fusion can already be observed in the earlier embryos where thoracic segments are not evenly spaced out, and is seen again in the hatchlings that display a
