# Supplementary figures and images for: Growth zone segmentation in the milkweed bug Oncopeltus fasciatus sheds light on the evolution of insect segmentation
Source: BMC Evol Biol. 2018 Nov 28;18:178. doi: 10.1186/s12862-018-1293-z (PMC6262967; doi:10.1186/s12862-018-1293-z)

Of-eve stain: wt

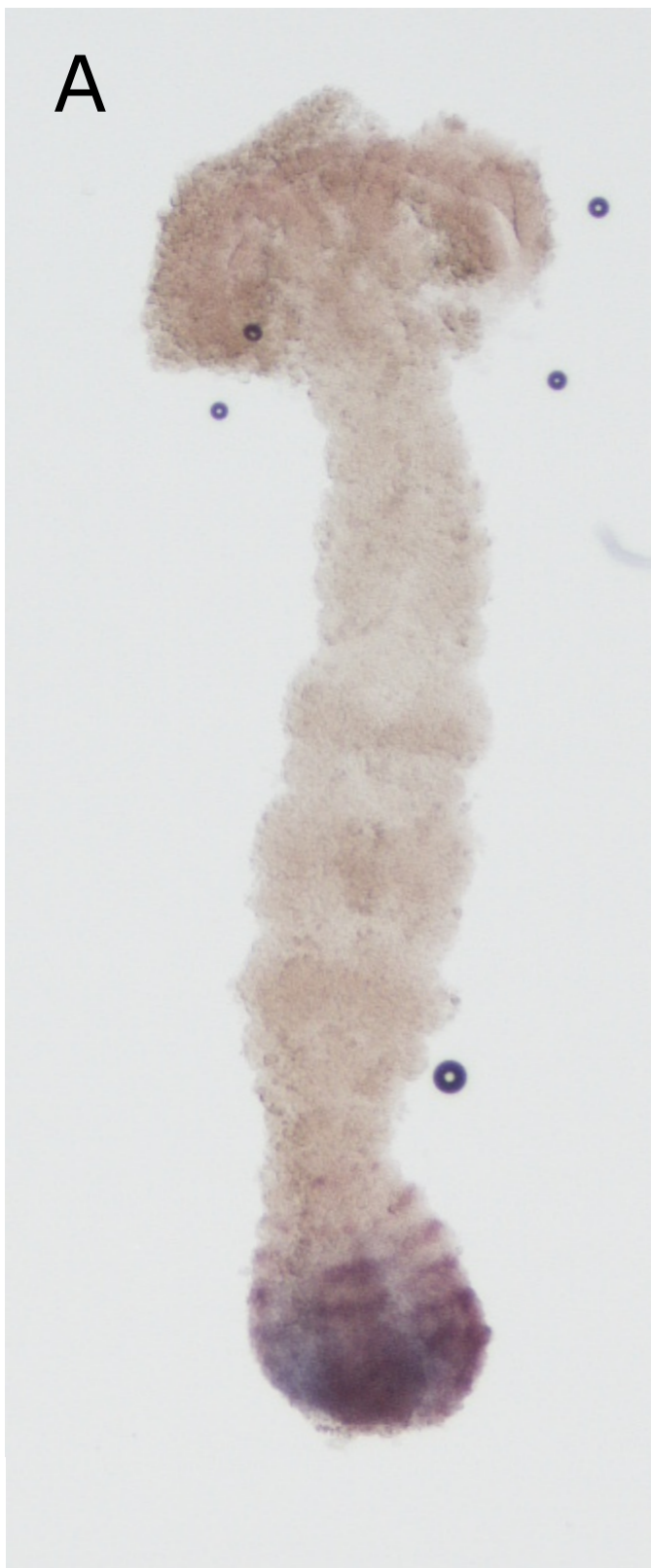

Of-odd<sup>RNAi</sup>

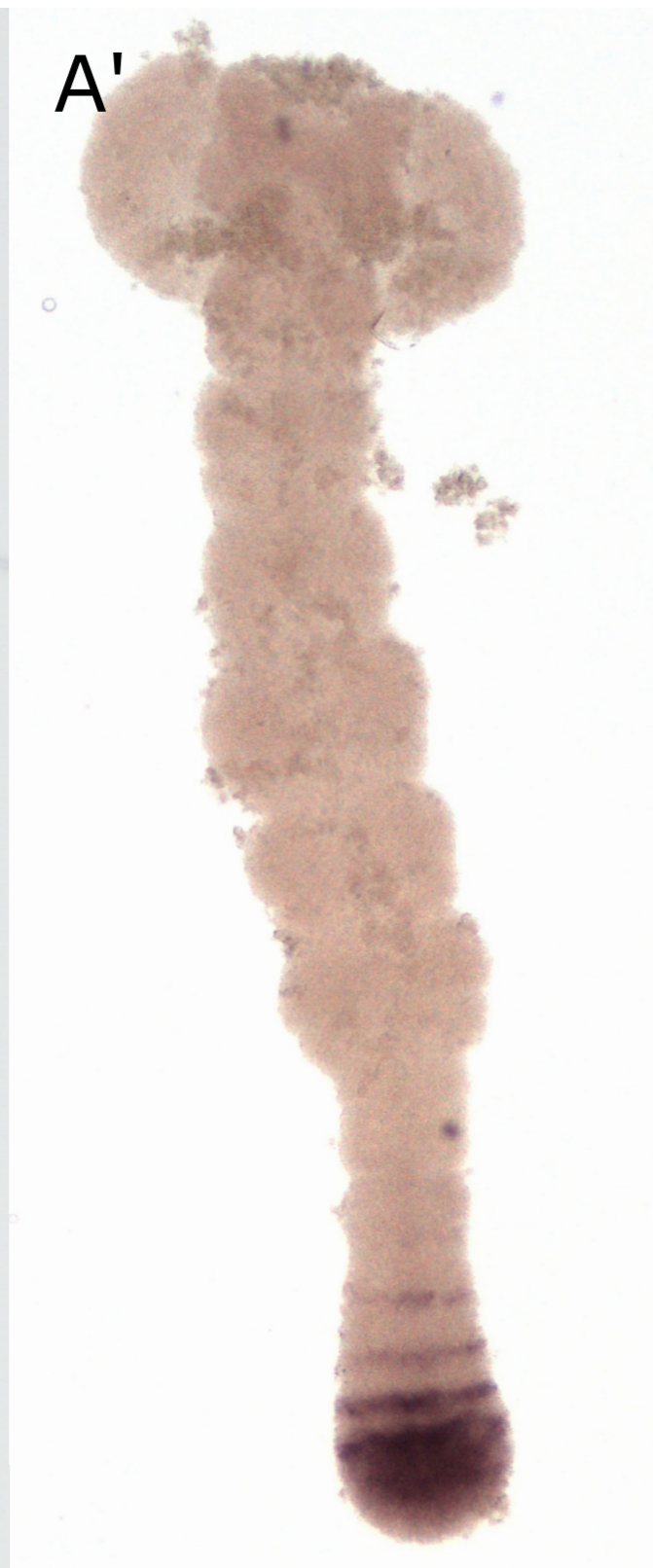

Of-slp<sup>RNAi</sup>

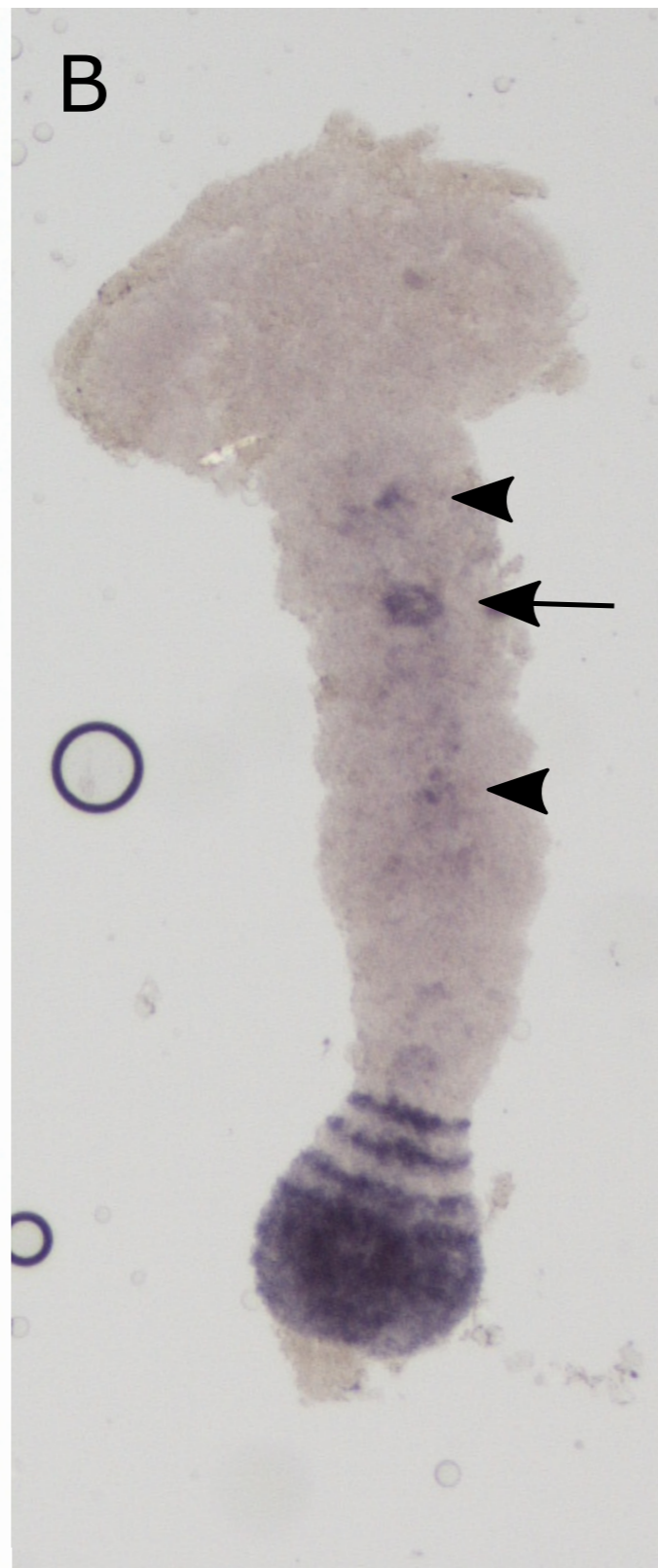

Of-hh<sup>RNAi</sup>

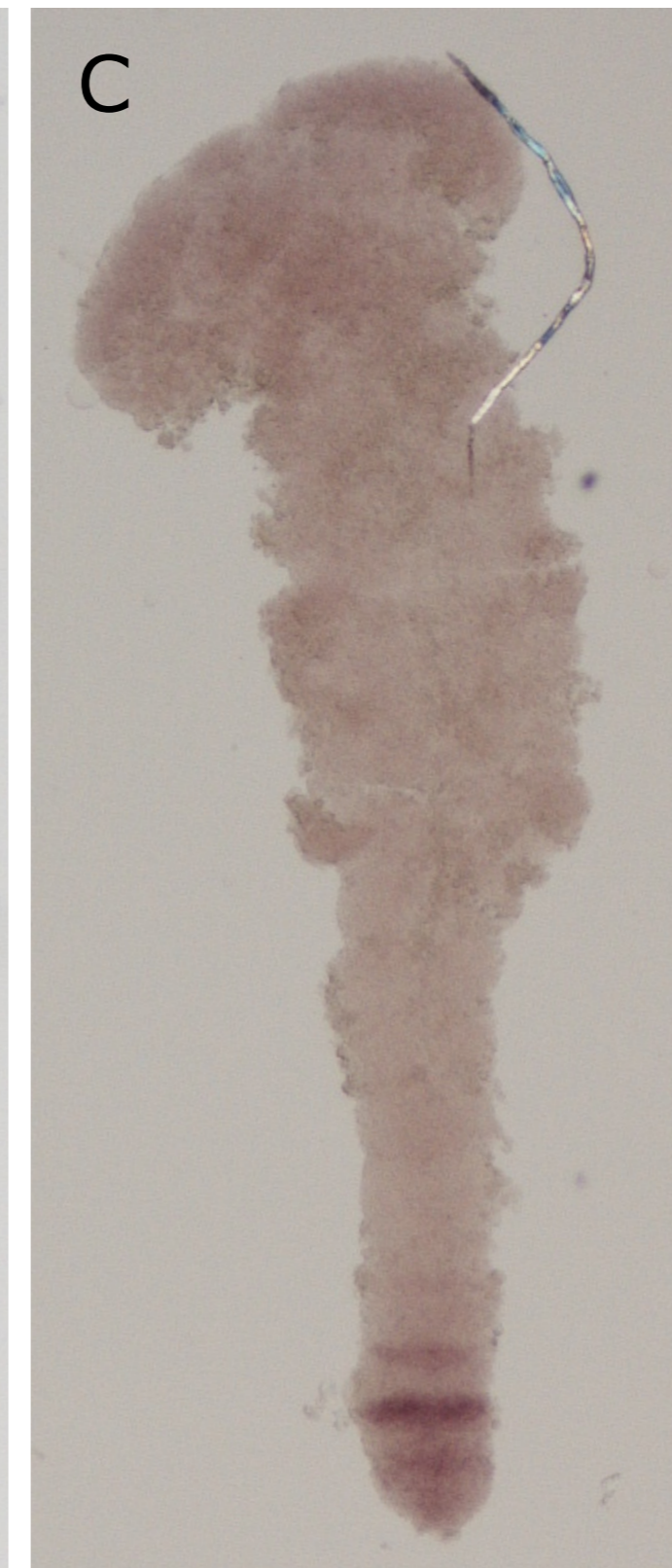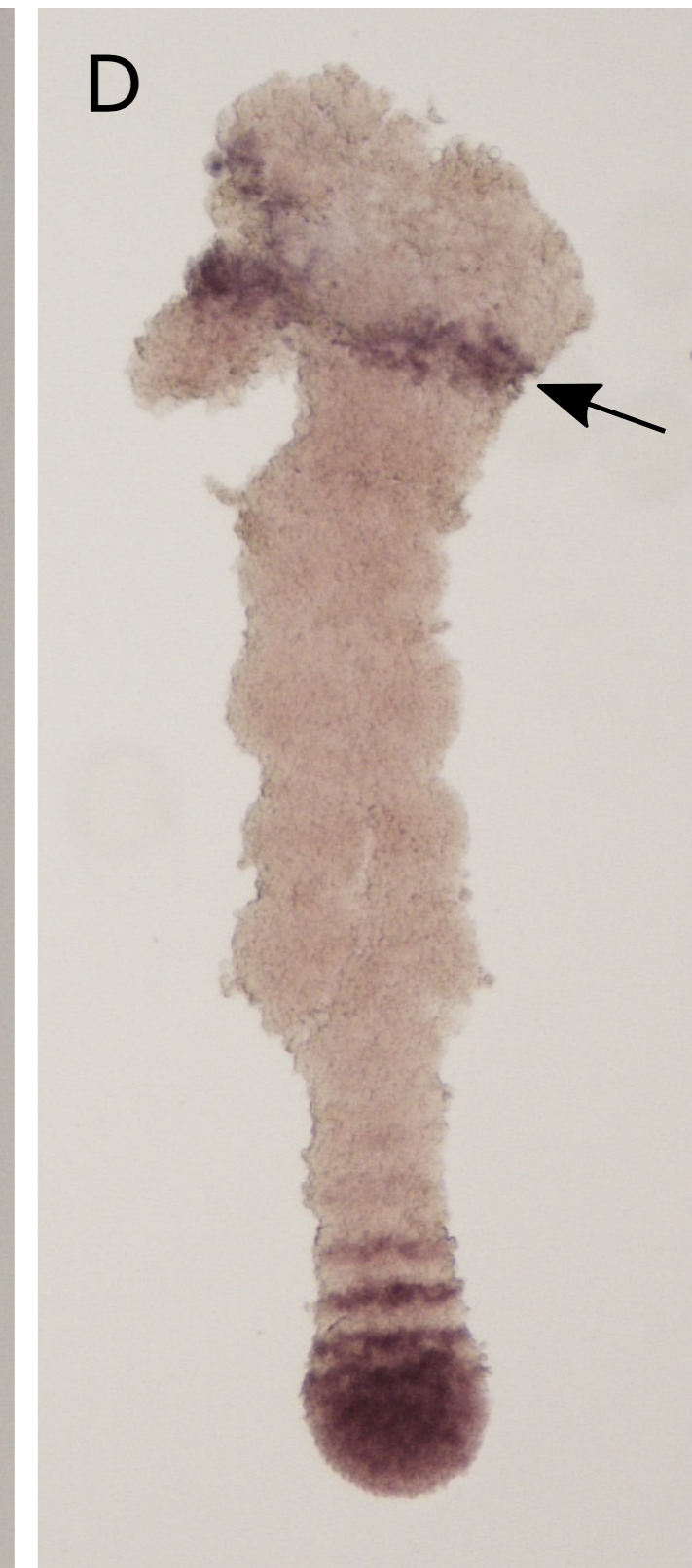

Supplement: Supplementary file 2 — eve staining of RNAi embryos. In addition to inv staining shown in the main text, eve staining was also performed on RNAi embryos. In relation to early and mid-stage WT embryos (A, A’) no substantial aberration of eve expression is seen in the GZ for any of the RNAi experiments. (B) in odd-RNAi embryos, eve is expressed ectopically in several medial patches in the thoracic and gnathal segments (arrow and arrowhead). When compared to the embryo in Fig. 7b, it seems that these points correspond to the areas where the thoracic segments fuse, or possibly to areas in the inv stripe that is more weakly expressed. (C) eve expression in slp-RNAi embryos is slightly out of the norm for the stage of the embryo, but this is mostly due to the abnormal general shape of the embryo. (D) hh-RNAi embryo displaying ectopic eve expression in the head, corresponding to the head phenotype displayed in Fig. 7d-d”. (PDF 1910 kb) [file 12862_2018_1293_MOESM2_ESM.pdf]
