# Supplementary material for: Growth zone segmentation in the milkweed bug Oncopeltus fasciatus sheds light on the evolution of insect segmentation
Source: BMC Evol Biol. 2018 Nov 28;18:178. doi: 10.1186/s12862-018-1293-z (PMC6262967; doi:10.1186/s12862-018-1293-z)
Supplement: Supplementary file 3 — Primers. Primers used to clone fragments for RNA in situ hybridization (upper table) and to clone dsRNA for RNAi experiments (lower table). (DOCX 14 kb) [file 12862_2018_1293_MOESM3_ESM.docx]

Primer sequences used to generate RNA probes for in situ hybridization:

| gene | F primer | R primer | GenBank accession number: |
| --- | --- | --- | --- |
| *hairy 3’* | ACCCTAGGTACTGTGCAGGT | CGTGCAGTCGCTTCTCCATA | MH090993 |
| *hh* | CTAGCGGTGGTCTGTTGGG | GTTTCAGCAACGCTCACTTCT | MH090996 |
| *opa* | CATCGGACTCAGTCTGCTGC | CTTGAGGTTCTCAGATCGGGC | MH090999 |
| *odd* | GCACAGTCAAGCTACCGTCT | ACTCTTTGGGCGAAGAAGGT | MH090997 |
| *prd* | CGAGCAGGCACAACACAAGA | ATGGCTGTTTCCACCCTCAA | MH091000 |
| *run* | GAGCTGATACAGACCGGCTC | ATAGGGCCTCCAAACGGTTG | MH091002 |
| *sob* | ATGGAGCCGGAGCTGGGTCG | CCTGAACGGCCGCCTAATCA | MH091003 |
| *slp* | TGAAGATGGAATCGGCACCC | TCTGAAAAACGAGCCCGACA | MH091004 |

Primer sequences used to generate double stranded RNA fragments for RNAi:

| gene | F primer | R primer |
| --- | --- | --- |
| *hh* 1^st^ fragment | TAATACGACTCACTATAGGGTACTACGAGACCAGAGCGC | TAATACGACTCACTATAGGGTCCTCGCAGCAAAGACAGTT |
| *hh* 2^nd^ fragment | TAATACGACTCACTATAGTTCAAGCAGCACGTACCCAA | TAATACGACTCACTATAGGCCAACATCCCGTACTTGGA |
| *slp* 1^st^ fragment | TAATACGACTCACTATAGGGAGAAGCATCCTCCCGGAAGA | TAATACGACTCACTATAGGGCACTTTCACGAAACAC |
| *slp* 2^nd^ fragment | TAATACGACTCACTATAGCTCGGAGGACGTGTTCATCG | TAATACGACTCACTATAGCTTGTAGATGGCAGGTCGGG |
| *odd* 1^st^ fragment | TAATACGACTCACTATAGGGCAGGAGGCTTGGTGAAGGAG | TAATACGACTCACTATAGGGCTTCTCCTTGCTGTGGAT |
| *odd* 2^nd^ fragment | TAATACGACTCACTATAGGAAGGGATTCTGCCAGTCCC | TAATACGACTCACTATAGACTCTTTGGGCGAAGAAGGT |
